# Basidiobolus: An unusual cause of lung abscess

**DOI:** 10.4103/0970-2113.63613

**Published:** 2010

**Authors:** Ravindran Chetambath, M. S. Deepa Sarma, K. P. Suraj, E. Jyothi, Safreena Mohammed, Beena J. Philomina, S. Ramadevi

**Affiliations:** *Department of Pulmonary Medicine, Medical College, Caicut, Kerala, India*; 1 *Department of Microbiology, Medical College, Caicut, Kerala, India*

**Keywords:** Basidiobolus, lung abscess, potassium iodide

## Abstract

Non-resolving pneumonia leading to lung abscess is always a challenge to the treating physician especially in a diabetic patient. Atypical radiological features of lung abscess should raise the suspicion of unusual organisms. This is a case report of a 42 year old diabetic male presented with features suggestive of lung abscess and multiple target organ damage. Subsequent work up revealed that the etiological agent is a rare fungus – Basidiobolus. To the best of our knowledge this is the first case of Basidiobolus lung abscess reported from India.

## CASE REPORT

A 42-year old male farmer from Wayanad, admitted to the Institute of chest disease, with complaints of high grade fever, cough and chest pain of three months duration. Cough was associated with expectoration of scanty white sputum. Chest pain was initially pleuritic but later became dull, aching and diffuse in nature. There was exertional dyspnoea which progressed to grade III during this period. He also had bilateral pitting pedal edema. There was no history of hemoptysis, hoarseness, headache, vomiting, abdominal pain or OSA symptom. He was a known diabetic patient since 20 years and, on admission, had symptoms of multiple target organ damage like diabetic neuropathy, retinopathy and nephropathy. There was no history of CAD or ATT in the past. He was recently detected to have hypertension. He was a smoker with a smoking score of 900. He was treated as a case of pneumonia from a local hospital and referred as there was no improvement.

On examination he had pallor, bilateral pedal edema and a blood pressure of 180/100 mmHg. There were generalized hyperkeratotic skin lesions with central necrosis over both lower limbs and back of chest. Some of them were simulating diabetic kyrle and some healed pyoderma. Respiratory system examination revealed a diagnosis of non-resolving pneumonia right upper lobe. He had minimal ascites.

**Figure 1:**
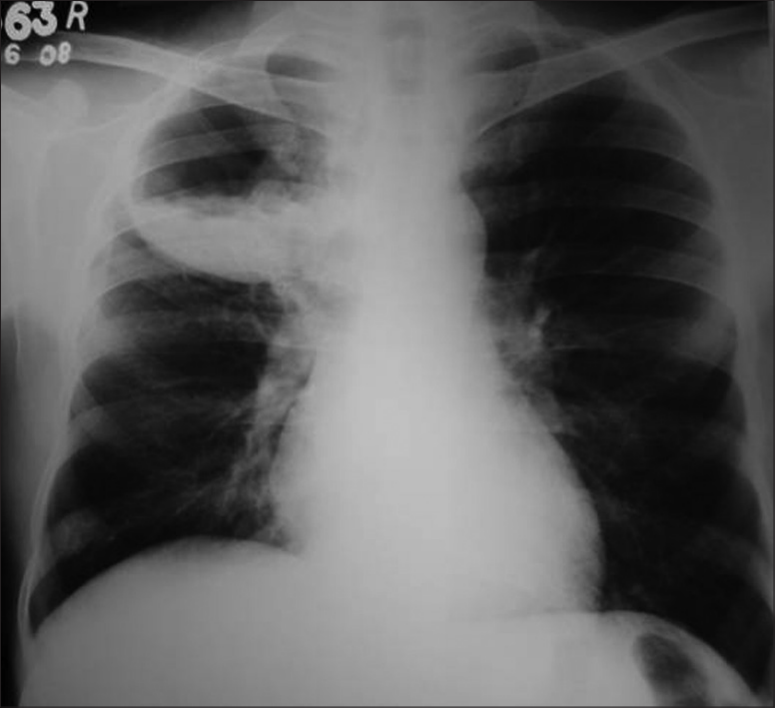
X-ray chest PA showing cavity with fluid level

**Figure 2:**
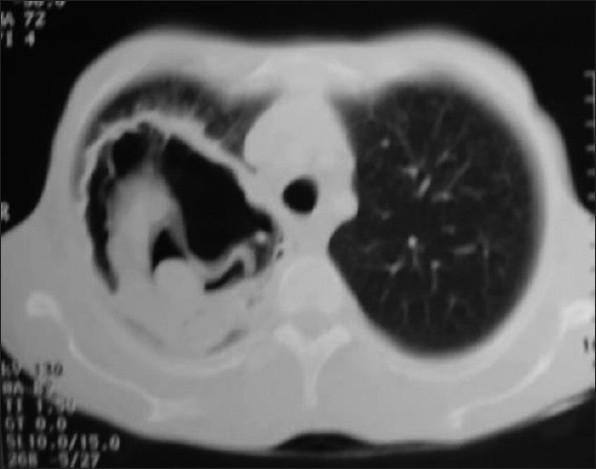
CT thorax showing cavity and its contents

Investigation revealed hemoglobin 7.7 g %, ESR 138 mm/first hour and mantoux test was non-reactive. His diabetic status and renal function were fluctuating throughout the hospital stay. Retroviral screening and sputum examination for AFB were negative. LFT were within normal limits. Chest X ray [Figure 1] showed a cavity in right upper zone with irregular inner wall, air fluid level and membranous projections from the wall. A lateral decubitus film demonstrated shifting of the fluid level with irregular cavity wall, and layers of air pockets in the cavity wall. As the X ray appearance was not typical of a lung abscess, possibilities like fungal infection, mycobacterial infection or hydatid cyst were considered in the differential diagnosis. CT thorax revealed fluid and solid attenuation areas mimicking a fungal ball as well as double layered cyst wall as in hydatid cyst [Figure 2]. USG abdomen showed changes of diabetic nephropathy, minimal ascites and mild hepatomegaly. There was no specific lesion in the liver to support a diagnosis of hydatid cyst. Echinococcal Ig G antibody was also negative.

**Figure 3:**
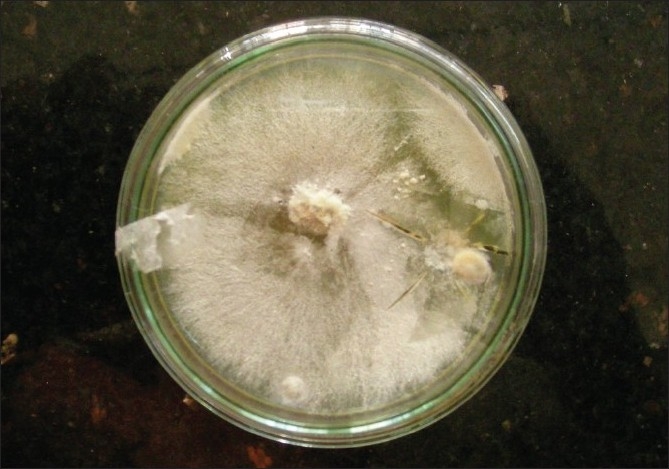
Culture media

**Figure 4:**
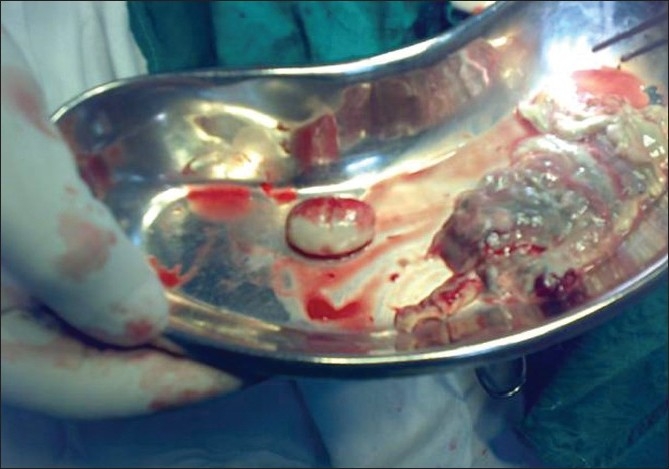
Surgical specimen

**Figure 5:**
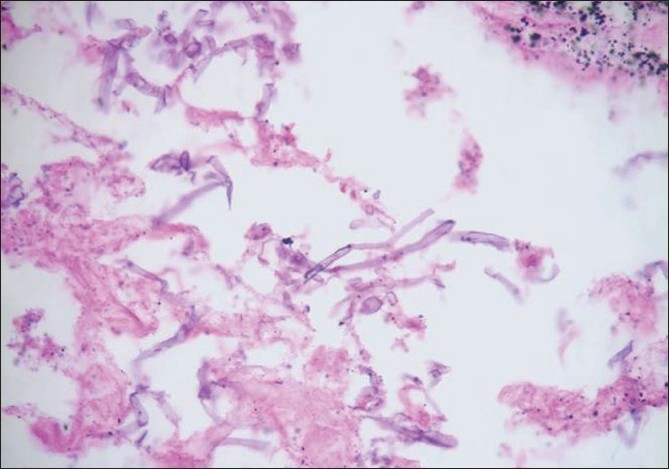
HPR showing fungal filaments

**Figure 6:**
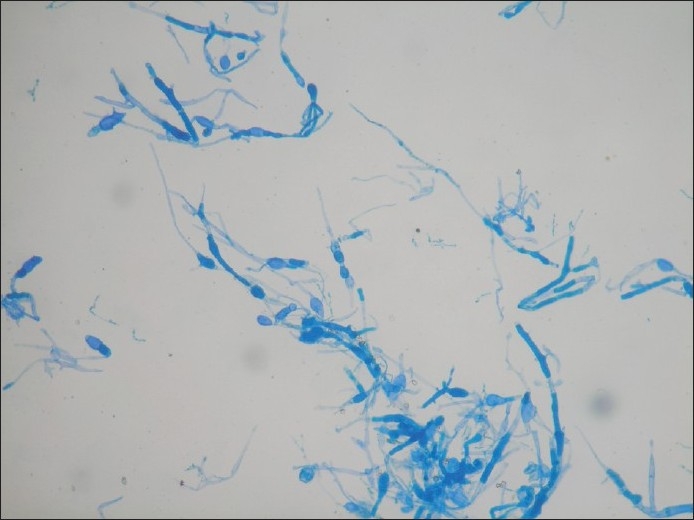
Fungal growth

**Figure 7:**
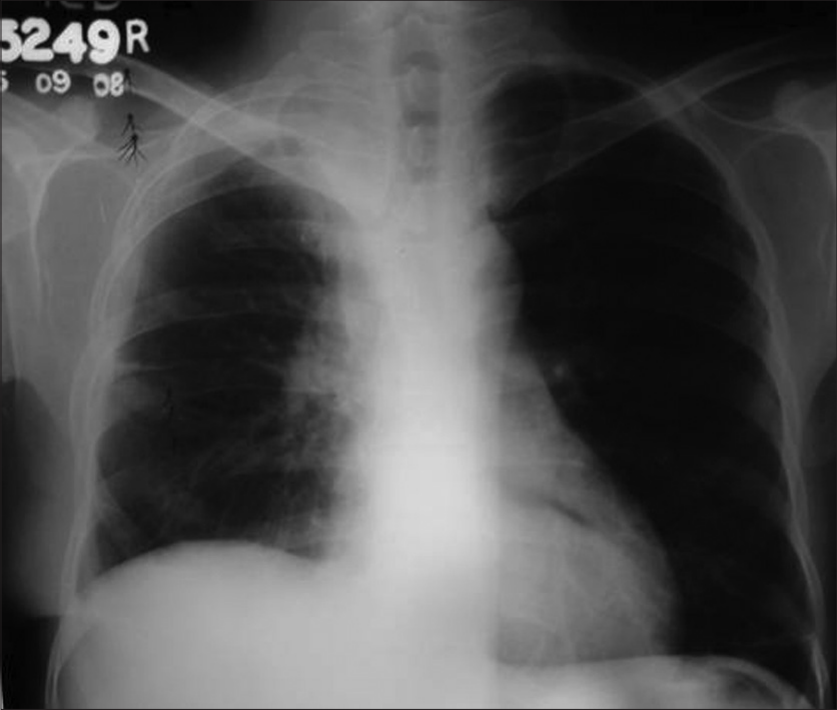
Postoperative X-ray

Bronchoscopy revealed mucosal edema and narrowing of right upper lobe apical bronchus from which creamy white secretions seen spurting with each cough. BAL was taken and sent for investigations. During this period, he was treated with broad spectrum antibiotics. As there was no improvement a trans-thoracic needle aspiration was done which yielded 10 ml of yellowish white fluid which was sent for investigation. The BAL as well as aspirated fluid specimen was mounted in lactophenol cotton blue which showed fungal growth which when incubated in Brain Heart infusion broth at room temperature showed the growth of a rare fungus, Basidiobolus [Figure 3]. Since potassium iodide was the drug of choice for this fungus, he was started on oral potassium iodide with 1 drop thrice daily and gradually increased to 10 drops thrice daily. He was also given cotrimoxazole as some cases reported an excellent response to this agent.[1,2] However his diabetic status worsened with the development of acute renal failure and hyperkalmia. KI was stopped temporally for control of renal failure and diabetes. The dose of insulin and parenteral fluids were carefully titrated. In order to achieve radical cure surgery was necessary as the patient's condition did not permit the use of the specific agent-KI. Hence patient was taken to cardio-thoracic surgery for resection after control of diabetes and renal function. Right upper lobectomy was done. The resected specimen showed a white ball of entangled mycelia [Figure 4]. Histopathological section showed filamentous aseptate fungi [Figure 5] and the report came as zygomycetes group of fungi and fungal culture confirmed the diagnosis of Basidiobolus ranarum [Figure 6]. Spendre–Hoeppli phenomenon was absent in this case and peripheral eosinophilia also is not demonstrated. Post-operatively, he was started on itraconazole 200 mg twice daily. A repeat CXR [Figure 7] six days after surgery showed near normal lung except for the presence of some amount of pleural thickening on the right side. There was good clinical improvement and he was discharged with itraconazole and other supportive measures.

## DISCUSSION

### Basidiobolus

This fungus, included in zygomycetes species, was described by Eidam in 1886. Basidiobolus is a filamentous fungus isolated from dung of amphibians, reptiles, and insectivorous bats, as well as wood lice, plant debris and soil. Even though it is cosmopolitan, human infections due to Basidiobolus are reported mostly from Africa, South America, and tropical Asia. Although three species of this organism are described (*Basidiobolus ranarum, Basidiobolus meristosporus*, and *Basidiobolus haptosporus*), isolates that are pathogenic to humans belong to a unique species, *Basidiobolus ranarum*. Infection is also called entomophthoromycosis basidiobolae or basidiobolomycosis. It can cause variety of clinical manifestations including subcutaneous, gastrointestinal, retroperitoneal and rarely pulmonary zygomycosis and occasionally an acute systemic illness similar to that caused by the mucorales.

Traumatic implantation or insect bite is probably the mode of entry like in other sub-cutaneous mycoses. Basidiobolus can cause rhinocerebral infections in hyperglycemic patients, indicating the probable route of entry via inhalation route.[3] It is characterized by its granulomatous nature and formation of hard, non-ulcerating subcutaneous masses at limbs, chest, back, and buttocks. But in the present case skin lesions were not typical for Basidiobolus infection and skin biopsy did not show any fungal filaments on histopathological examination. Culture with skin biopsy specimen was not done in this case. Systemic infection is rare. Pulmonary and retroperitoneal infections are reported in immuno-competent host and opportunistic infections in immuno-compromised host.[4] There is a case report of pulmonary basidiobolomycosis, in an young immunocompetent women who had presented with eosinophilia and lung infiltrates. She subsequently died and diagnosis of disseminated basidiobolomycosis was made on the basis of histological features at autopsy.[5]

Cases of sub-cutaneous zygomycosis caused by basidiobolus are reported from south India. Gastrointestinal infections due to Basidiobolus ranarum have also been reported in man.[6] This is one of the differential diagnoses of inflammatory bowel diseases in endemic areas. Presentation as pseudo-tumour and invasive retroperitoneal infection was also reported.[7]

Basidiobolus grows on potato glucose agar producing a heaped up or radially folded, grayish brown colony covered by fine, white, powdery surface mycelium. Germinated conidia form nearby satellite colonies. This organism usually produces asexual spores which germinate to form capilliconidiophore. The Splendore-Hoeppli phenomenon describes eosinophilic and pseudomycotic structures, composed of necrotic debris and immunoglobulin, which may not be present in all cases. Histo-pathological sections show non-parallel randomly branching hyphae. There is a chronic inflammatory response with a predominance of eosinophils.[8] Basidiobolus ranarum infections may be angio-invasive in immuno-compromised hosts.

An immuno-diffusion test was described using culture filtrate antigen from Basidiobolus ranarum. This can be used to monitor treatment response in patients infected with this organism.[9] Elevated IgG, IgM were also demonstrated by Elisa.[7]

### Treatment

No single drug was proven to be effective. As the disease is rare no randomized control studies are available to compare the treatment regimen. Infections due to Basidiobolus are difficult to treat. Systemic potassium iodide solution and trimethoprim-sulfamethoxazole are usually preferred. To be efficacious KI should given for three months one/ two grams per day. In most cases skin lesions completely subsided with oral KI.[10] KI is successful in subcutaneous infection but data is lacking for visceral infection. Oral ketoconazole, fluconazole or itraconazole may be of help in some cases.[2,11] Generally combination drugs are preferred and Amphotericin B has practically no significant efficacy. Usually infection caused by Basidiobolus is not curative with surgery. However, if lesions are localized, as in this case, the result of surgery is excellent. In this case there was significant initial response with itraconazole post-operatively, His diabetic status was well controlled and kept under follow up.
